# Clinical impact of non-lying time on hospital-associated functional decline in older patients undergoing transcatheter aortic valve implantation

**DOI:** 10.1007/s00380-023-02326-w

**Published:** 2023-10-16

**Authors:** Yuji Kono, Masahiko Mukaino, Yushi Ozawa, Koji Mizutani, Yuki Senju, Takayuki Ogasawara, Masumi Yamaguchi, Takashi Muramatsu, Hideo Izawa, Yohei Otaka

**Affiliations:** 1 Department of Rehabilitation, Fujita Health University Hospital, Toyoake, Japan; 2 Department of Rehabilitation Medicine I, School of Medicine, Fujita Health University, 1-98 Dengakugakubo, Kutsukake-Cho, Toyoake, Aichi 470-1192 Japan; 3 NTT Basic Research Laboratories and Bio-Medical Informatics Research Center, NTT Corporation, Atsugi, Japan; 4 Department of Cardiology, School of Medicine, Fujita Health University, Toyoake, Japan

**Keywords:** Transcatheter aortic valve implantation, Hospital-associated functional decline, Older patients, Non-lying time

## Abstract

The purposes of the present study were: (1) to investigate the relationship between hospital-associated functional decline (HAFD) and non-lying time and (2) to clarify the optimal cut-off value for non-lying time associated with HAFD in older patients undergoing transcatheter aortic valve implantation (TAVI). From January 2021 to December 2022, patients admitted to a university hospital who underwent trans-femoral TAVI were consecutively recruited. We measured short physical performance battery (SPPB) pre and post-TAVI, and non-lying time from post-operative days 3–5. HAFD was defined as at least 1 point decrease in SPPB during pre and post-TAVI. Among 75 patients (47 female, mean age of 84.5 years) enrolled, 14 patients were classified as having HAFD. Non-lying time was significantly shorter in the HAFD group than in the non-HAFD group (371 min vs. 539 min, *P* < 0.001). Receiver-operating characteristic analysis determined an optimal cut-off value of 477 min for differentiating the patients more likely to experience HAFD (sensitivity, 75%; specificity, 92%; area under the curve, 0.798). The non-lying time could be one of the associated factors of HAFD in older patients with TAVI. Non-lying time of about 480 min (8 h) during hospitalization may be an initial target for preventing HAFD.

## Introduction

Prolonged bed rest plays a major role in deconditioning, resulting in significant muscle wasting and the development of functional decline in acute care settings [1]. Hospital-acquired functional decline (HAFD), which refers to either a new or worsened functional decline during hospitalization, is a widely accepted conception among older hospitalized patients [2]. HAFD develops in at least 30% of hospitalized older patients, and closely associated with poor prognosis among cardiac patients [2].

Over the last twenty years, transcatheter aortic valve implantation (TAVI) has become the standard treatment for patients with severe aortic stenosis in worldwide [3]. Older age, lower renal or liver function, dementia and lower physical function or activities of daily living, so-called frailty patients, are common indication criteria for TAVI recommended in the existing guidelines [4]. Based on the previous findings, it is suggested that the patient undergoing TAVI is more likely to get HAFD. Previous studies reported that prevalence rate of HAFD is 20–30% and HAFD is closely associated with post-discharge heart failure readmission and cardiac death [5, 6]. Thus, preventing HAFD is a primary goal of rehabilitative intervention for patients undergoing TAVI.

Several previous studies demonstrated that lower physical activity, especially daily step counts, and shorter rehabilitation time is possible risk factor for HAFD [7, 8]. The link between the amount of physical activity and HAFD implies that early mobilization and enhancing physical activity during hospitalization may be effective in preventing HAFD. However, many older hospitalized patients spend most of their time in bed with lying [9]. We hypothesized that non-lying time, in addition to other clinical parameters, would be associated with HAFD among older hospitalized patients undergoing TAVI.

The aims of the present study were: (1) to investigate the relationship between HAFD and non- lying time and (2) to clarify the optimal cut-off value for non-lying time associated with HAFD in older patients with TAVI.

## Materials and methods

### Study design and setting

The study was a prospective cohort study at a university hospital in Japan. This study complied with the principles of the Declaration of Helsinki regarding investigations in humans and was approved by the Research Ethics Committee of Fujita Health University (Approval No: HM17-220).

### Participants

From January 2021 to December 2022, we enrolled consecutive patients who had been admitted to Fujita Health University Hospital to underwent trans-femoral TAVI were recruited. Patients were excluded if they could not walk independently, had severe dementia which unable to understand research purpose and posture monitoring, had severe post-operative complication which need to restrict physical activity, or if they did not wish to participate. All the participants provided written informed consent prior to the study.

### Data collection

Age, gender, body mass index (BMI), and pharmacotherapy for each patient on admission were obtained as a background. We obtained serum hemoglobin, serum albumin, N-terminal pro B-type natriuretic peptide, and the estimated glomerular filtration rate from the results of a blood investigation and additionally performed echocardiography to obtain the left ventricular ejection fraction (LVEF).

We measured following physical function tests at 1 day before TAVI. Handgrip strength was measured by a grip strength dynamometer. The participants were asked to sit with their wrist in a neutral position and elbow extended to 0°. Grip strength was measured twice for each hand, and the highest value was used for the analysis. Comfortable gait speed was evaluated by the 10-m usual walk test; subjects were requested to walk at their usual pace for 14 m, of which the middle 10 m was timed. The test was completed twice, and the speed in the faster trial was used for analysis. In addition, pre-operative physical frailty was assessed with the Japanese version of the Cardiovascular Health Study criteria [10], which has reported validity and reliability in Japanese older heart failure patients [11]. These criteria consist of five items: (1) Have you lost two kilograms of weight or more in the past six months?; (2) Do you engage in moderate levels of physical exercise or sports aimed to promote health? Do you engage in low levels of physical exercise aimed to promote health? (no to both questions = 1); (3) In the past two weeks, have you felt tired without cause?; (4) grip strength < 26 kg in men or < 18 kg in women; and (5) gait speed of < 1.0 m/s. If three or more of the above five items were applied, the patient was defined as frailty. We also measured Japanese version of Montreal Cognitive Assessment (MoCA-J) as cognitive function and Mini Nutritional Assessment Short-Form (MNA-SF) as nutritional status.

The Short Physical Performance Battery (SPPB) was evaluated as a parameter of total physical functional, and we measured at 1 day before TAVI and at 1-week post-TAVI. The SPPB is a highly standardized geriatric physical functioning test that consists of tests for balance, gait, strength, and endurance [12]. The balance test evaluated the ability to stand with both feet together side-by-side in a semi-tandem and tandem position. The gait test assessed the time to walk 4 m, performed at the patient’s usual pace. The 5-time chair-standing test measured the time to rise from a chair 5 times consecutively with arms folded across the chest as quickly as possible. The total SPPB scores ranged from 0 to 12 points, and higher scores indicated better physical functioning status.

### Recognition of lying or non-lying posture

The hitoe system is equipped with an accelerometer for patient posture monitoring, which operates with a measurement range of ± 4G and a sampling frequency of 25 Hz. The system processes these data every minute to compute the patient’s posture, based on the angle or declination of the accelerometer relative to the direction of the measured gravitational acceleration in the sagittal plane of the patient’s trunk [13, 14]. Using the angle and a designated threshold, postures are initially classified as ‘lying’ or ‘non-lying’. The reliability of this posture recognition approach has been reported in both healthy subjects [13] and clinical patients [15].

### Definition of HAFD

HAFD was defined by a decrease in at least 1 point on the SPPB one-week post-TAVI compared to the score on 1 day before TAVI, because a change in SPPB score of 1.0 point was considered as a minimal clinically important difference in HF patients [16].

### Statistical analyses

Continuous variables were expressed as the median (interquartile range [IQR]), and category variables as number and percentage. We divided patients into two groups according to the presence or absence of HAFD (the HAFD group and the non-HAFD group). To clarify the difference in characteristics between groups, clinical variables were compared by the Mann–Whitney *U* test for continuous variables and chi-square test or Fisher’s exact test for categorical variables. In addition, multivariable logistic regression analyses were used to evaluate the independent association between non-lying time and HAFD. The multivariate regression analysis was performed predictive models based on preexisting potential and confounding factors.

Receiver-operating characteristic (ROC) curves were constructed, and the area under the curve was analyzed to confirm validity of non-lying time for predicting HAFD, and the Youden Index was calculated to obtain the optimal cut-off value for distinguish HAFD. Negative and positive predictive value were estimated to determine the usefulness of clinical variables.

All analyses were performed using SPSS 26.0 software package (SPSS Inc., Tokyo, Japan), with a p value of less than 0.05 being considered statistically significant.

## Results

### Patient characteristics

**Fig. 1:**
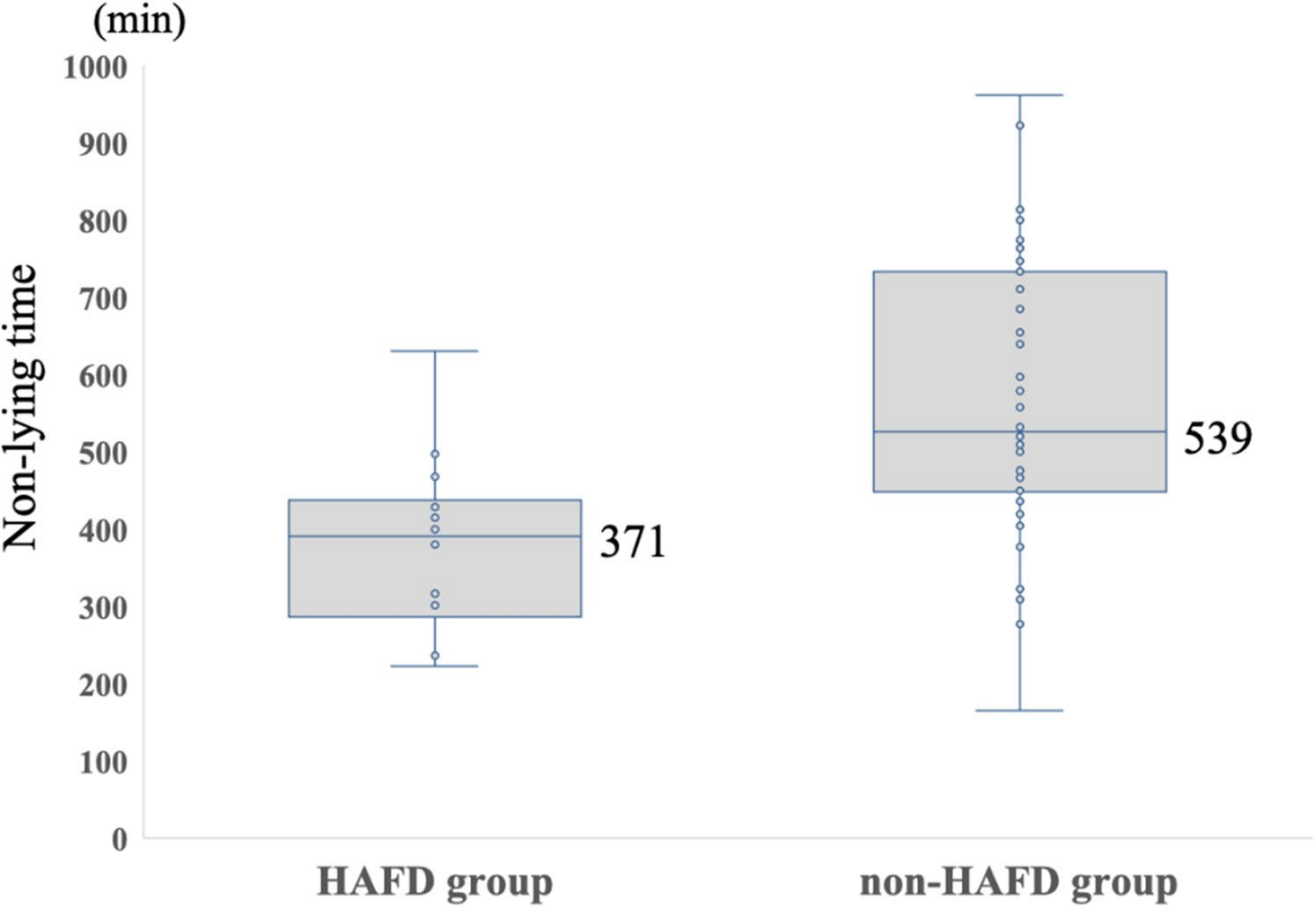
Comparison of non-lying time between groups

Of a total of 82 patients, 7 patients were excluded because of post-operative complication (atrioventricular block 5, stroke 1, aortic dissection 1), and we had no patients who could not walk independently or severe dementia. Finally, we enrolled 75 patients (median age 85 years; 47 women), and Table 1 shows a comparison of clinical characteristics between the HAFD groups (*n* = 14) and the non-HAFD group (*n* = 61). All patients had no history of cerebrovascular disease. Post-operative SPPB score was significantly lower in the HAFD group than in the non-HAFD group (7 vs 10, *p* = 0.002). There were no significant differences between the groups in all the variables, and all patients underwent trans-femoral approach. Non-lying time was significantly shorter in the HAFD group than in the non-HAFD group (371 min vs. 539 min, *P* < 0.001) (Fig. 1).

**Table 1:** Demographic and baseline clinical characteristics of participants

	Total (*N* = 75)	HAFD group (*n* = 14)	Non-HAFD group (*n* = 61)	*P*
Age, years	85 (81, 88)	87 (83, 89)	86 (82, 91)	0.886
Gender, female, *n* (%)	47 (62.6)	9 (64.3)	38 (62.2)	0.885
BMI, kg/m^2^	22.3 (19.9, 23.6)	22.8 (20.3, 23.1)	22.2 (18.9, 23.7)	0.495
NYHA class III or IV, *n* (%)	12 (16)	4 (28)	8 (13)	0.534
Post-operative hospital stay, days	9 (8, 10)	9 (8, 11)	9 (8, 9)	0.811
Post-operative days of start walking, days	2 (2, 2)	2 (2, 2)	2 (2, 2)	1.000
Total rehabilitation time, minute	120 (100, 120)	120 (100, 120)	120 (100, 120)	0.912
MoCA-J, points	25 (22, 28)	26 (22, 28)	26 (25, 27)	0.133
MNA-SF, points	13 (12, 14)	13 (11, 13)	13 (11, 13)	0.269
*Comorbidities* *n* (%)				
Hypertension	56 (72)	10 (75)	46 (75)	0.446
Diabetes	18 (24)	4 (28)	14 (23)	0.785
Dyslipidemia	35 (47)	6 (42)	29 (48)	0.165
CKD	53 (71)	10 (71)	43 (70)	0.433
History of Heart failure	14 (19)	3 (21)	11 (18)	0.212
Serum hemoglobin, g/dl	11.6 (10.6, 12.7)	11.3 (10.3, 12.8)	11.8 (10.6, 12.7)	0.546
Serum albumin, g/dl	3.6 (3.3, 3.8)	3.4 (3.2, 3.5)	3.6 (3.5, 3.7)	0.133
Serum creatinine, g/dl	0.83 (0.72, 1.00)	0.82 (0.71, 0.93)	0.84 (0.79, 1.09)	0.470
eGFR, ml/min/1.73m^2^	45.3 (40.5, 62.1)	41.4 (39.6, 61.7)	47.1 (38.6, 62.1)	0.433
NT-proBNP, pg/ml	931 (341, 1612)	1189 (414, 2171)	714 (336, 1540)	0.727
LVEF, %	60 (57, 63)	60 (58, 63)	60 (57, 63)	0.774
Grip strength, kg	19.8 (16.6, 22.8)	17.5 (16.3, 22.8)	19.8 (14.6, 21.2)	0.164
Comfortable gait speed, m/s	0.99 (0.85, 1.27)	0.93 (0.74, 1.06)	1.01 (0.79, 1.19)	0.316
Pre-operative SPPB score, points	10 (9, 12)	10 (9, 11)	10 (8, 11)	0.900
Pre-operative frailty, n (%)	35 (47)	8 (57)	27 (44)	0.138

Values are presented as median (interquartile range) or number (%)

*BMI* body mass index, *MMSE* NYHA, New York Haret Association, *MoCA-J* Japanese version of the Montreal Cognitive Assessment, *MNA* Mini-mental state examination, *CKD* chronic kidney disease, *NT-pro BNP* N-terminal pro-brain natriuretic peptide, *LVEF* left ventricular ejection fraction, *SPPB* Short physical performance battery

### Multivariable analysis

The independent association between non-lying time and HAFD was analyzed by a covariate adjustment method using propensity scores because of the small incidence of HAFD. To calculate the propensity score, factors that have been reported to be associated with HAFD were selected from the pre-operative variables, and combinations of factors that predict HAFD were examined by a multivariable logistic regression analysis. After adjustment for propensity score calculated by the prediction model consisting of age, gender, serum albumin, grip strength, and pre-operative frailty, non-lying time was selected as independent associated factor of HAFD (Table 2).

**Table 2:** Multivariable logistic regression analysis for HAFD, with non-lying time as an independent factor

	B	SE	Wald	P	OR	95%CI	
						Lower	Upper
Non-lying time	– 0.008	0.003	9.459	0.002	0.992	0.986	0.997
Propensity score	6.459	2.784	5.384	0.02	638.369	2.727	149,461.061
(Constant)	1.003	1.184	0.717	0.397	2.725		

Propensity score: age, gender, serum albumin, grip strength, pre-operative frailty

*SE* Standard error, *OR* Odds ratio, *CI* Confidential interval

### ROC analyses

**Fig. 2:**
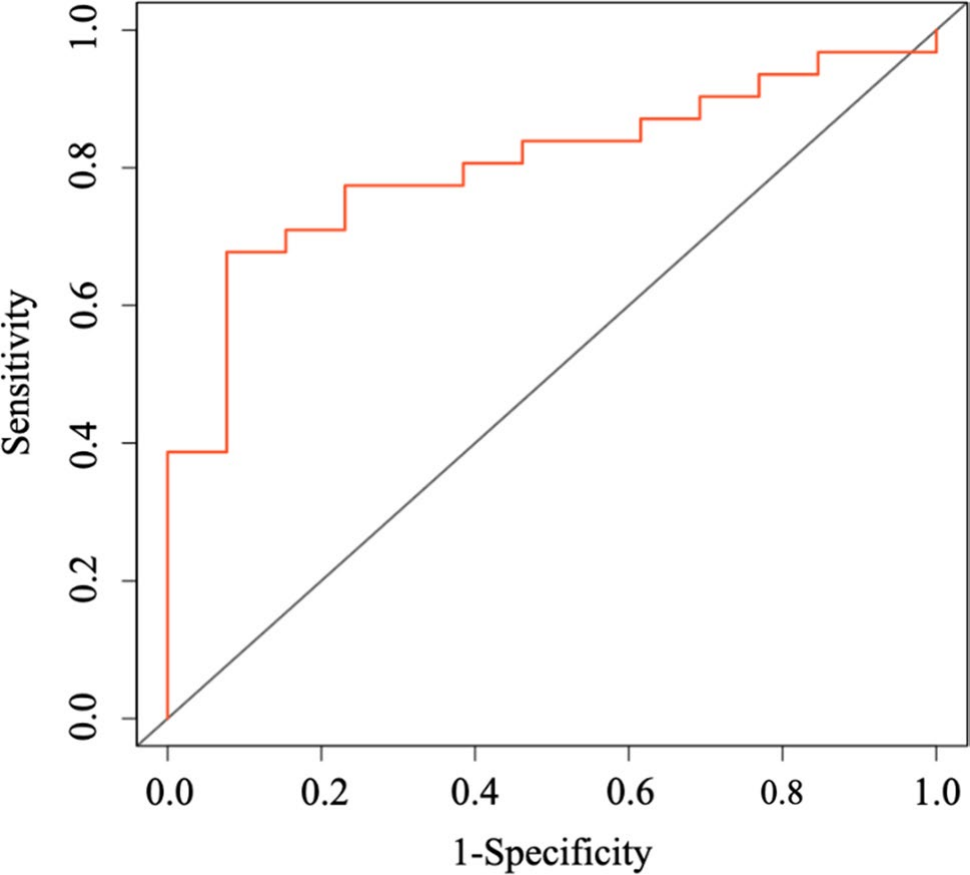
Receiver-operating characteristic (ROC) analysis of the non-bedrest time (minutes) as a predictor of HAFD. The optimal cut-off value for distinguishing subjects who would experience HAFD was 477 min. This cut-off gave a sensitivity of 75.2% and a specificity of 92.3%; the area under the curve was 0.798 (95%CI 0.683–0.912, *p* = 0.001)

ROC curve analysis with the HAFD as the outcome identified a value of 477 min as the optimal cut-off level predictive of HAFD. Using this cut-off gave a sensitivity of 75.2% and a specificity of 92.3%; the area under the curve was 0.798 (95%CI 0.683–0.912, *p* = 0.001) (Fig. 2). The positive and negative predictive values of HAFD less than 477 min were 73.3% and 88.9%, respectively.

## Discussion

The main finding of this study was that, for older patients who underwent TAVI, the non-lying time was a significant predictive parameter of HAFD. The analysis also showed that 477 min (about 8 h) the optimal cut-off value, and the patients who achieved non-lying time more than 477 min were significantly less likely to be HAFD. To the best of our knowledge, this was the first study to demonstrate a relationship between non-lying time and incidence of HAFD and to establish a cut-off value for distinguishing those at greater risk.

The result of this study shows that the prevalence rate of HAFD 18.6% and was consistent with the previous studies that reported 20 to 30% of prevalence rate [5, 6] in the population with similar age, gender, history of heart failure, and other complications. Thus, it is suggested that our sample is likely to reflect the general TAVI patient population, and our data also identified that HAFD is common even in elective post-TAVI patients. It is well established that impaired physical function predicts future disability and mortality in older patients in general [17]. Therefore, maintenance of physical function during acute phase hospitalization is also one of the major rehabilitation outcomes for older patients with TAVI.

The result of multivariable analysis indicated that non-lying time was selected as independent related factor of the HAFD after adjustment for propensity score. Although an association between HAFD and in-hospital physical activity including step count [7] and rehabilitation time [8] has previously been reported in heart failure patients, the association with non-lying time has not been addressed. A possible explanation for this finding may be that sitting/standing posture reduced the risk of muscle weakness including iatrogenic sarcopenia, a major hospital-acquired comorbidity caused by excessive bed rest in an acute phase clinical setting. Previous studies have reported that bed rest is closely associated with rapid muscle weakness and muscle atrophy [18], and early mobilization had favorable effects on physical function at discharge [19]. In the present study, all patients were able to start walking training from the post-operative day 2 and total rehabilitation time during hospitalization was same between groups. Thus, our results suggested that the non-lying time might be a possible risk factor of HAFD, especially in older TAVI patients.

In the present study, we clarified that the optimal cut-off value of non-lying time for differentiating between the HAFD and the non-HAFD groups was 477 min, and the area under the curve was 0.798, indicating excellent ability to distinguish between the groups [20]. Indeed, non-lying time less than 477 min had an 89% negative predictive value in the present study, suggesting that non-lying time more than 477 min could be used to stratify patients at low risk of developing HAFD. Developing therapeutic interventions to prevent HAFD for older TAVI patients remains a challenge. Although we cannot demonstrate the cause–effect relationship between non-lying time and HAFD, it is suggested that non-lying time more than 477 min (about 8 h) might be a practical target in managing the activity level of the patients after TAVI to reduce the occurrence of HAFD.

Previous reports have identified several prognostic parameters of post-TAVI patients. Some clinical markers, such as blood laboratory test and nutritional status, and physical function [21–23], have provided a basis for prognostic risk stratification; however, specific intervention strategy to prevent HAFD was not proposed. In contrast, the non-lying time is easily evaluated and is easy to understand even for the patients and all medical staff. Therefore, it could play an important role in patients management programs for preventing HAFD for not only post-TAVI patients but also older medical hospitalized patients.

This study had some potential limitations. First, it was based on a single-center registry of patients with TAVI and the results may not necessarily be generalized to groups with dissimilar demographic characteristics. Next, although we confirmed independent association between HAFD and non-lying time by multivariable analysis, we could not adequately adjust potential confounding factors, including pre-operative physical function and physical activity, because of small sample size. Lastly, this study was designed as observational study, so the level of evidence was lower than that provided an interventional study. Thus, we could not determine the effectiveness of non-lying time more than 477 min (8 h) on preventing HAFD, further interventional study is required to confirm the efficacy of staying non-lying position. However, the results of the present study provided novel information that non-lying time could be a key component of acute phase treatment.

## Conclusions

In conclusion, our results showed that the non-lying time might be one of the potential related factors for HAFD in older post-TAVI patients. Non-lying time of more than 477 min (8 h) might be an initial target for acute phase post-TAVI management.
